# Evaluation of sudden cardiac death in hypertrophic cardiomyopathy

**DOI:** 10.1186/s44348-025-00054-5

**Published:** 2025-10-16

**Authors:** Sang Gon Yoon, Geu-Ru Hong

**Affiliations:** Division of Cardiology, Severance Cardiovascular Hospital, Yonsei University College of Medicine, Seoul, Republic of Korea

**Keywords:** Hypertrophic cardiomyopathy, Sudden cardiac death, Pathophysiology, Etiology, Risk factors

## Abstract

Hypertrophic cardiomyopathy has become a highly manageable condition due to recent therapeutic advances that have significantly reduced its overall mortality rate. However, sudden cardiac death continues to be a critical and unsolved threat, particularly in younger patients and competitive athletes. Even after recent updates to guidelines on sudden cardiac death risk evaluation in hypertrophic cardiomyopathy, new clinical evidence continues to emerge, further enriching our understanding of risk stratification and management. In this review, we summarize current research findings and explore recent advances to provide insights into future directions in the treatment of hypertrophic cardiomyopathy.

## Introduction

Hypertrophic cardiomyopathy (HCM), a globally prevalent primary cardiac disease with a genetic basis, has become a highly manageable condition due to recent advances in treatment [1, 2]. Since the initial pathologic insights by Teare [1, 3] and comprehensive clinical descriptions by Braunwauld et al. [4] in the early 1960 s, great progress has been achieved in the diagnosis and management of HCM. Current therapeutic strategies that target adverse pathways and can be tailored to individuals of all ages have significantly reduced annual HCM mortality, from 6% reported in the 1960 s to 0.5%, which is currently one of the lowest of all major disease-related risks (e.g., cancer, neurological disorders, congestive heart failure) [1, 5–8]. However, in adolescents and young adults, particularly in competitive athletes, HCM remains the leading cause of sudden cardiac death (SCD) [1, 9–12].

In the context of SCD, the implantable cardioverter-defibrillator (ICD) plays a central role in the management of HCM. A landmark study published in the *New England Journal of Medicine* in 2000 demonstrated that ventricular tachyarrhythmias were the primary cause of cardiac arrest in HCM patients, and that ICDs could reliably detect and terminate these life-threatening arrhythmias [13]. Consequently, ICD implantation has become a critical treatment strategy for preventing SCD in high-risk HCM patients [13]. Before the advent of the ICD, high-risk patients were administered cardioactive pharmacological agents (e.g., β-blockers and calcium channel blockers and antiarrhythmic agents such as amiodarone) for SCD prophylaxis [9].

As more patients are being diagnosed with HCM and as treatment strategies for HCM improve, identifying high-risk individuals has become more important. In this review, we focus on the pathophysiology, risk factors, and most recent advances in management strategies for SCD in patients with HCM.

### Pathophysiology of SCD in HCM

**Fig. 1:**
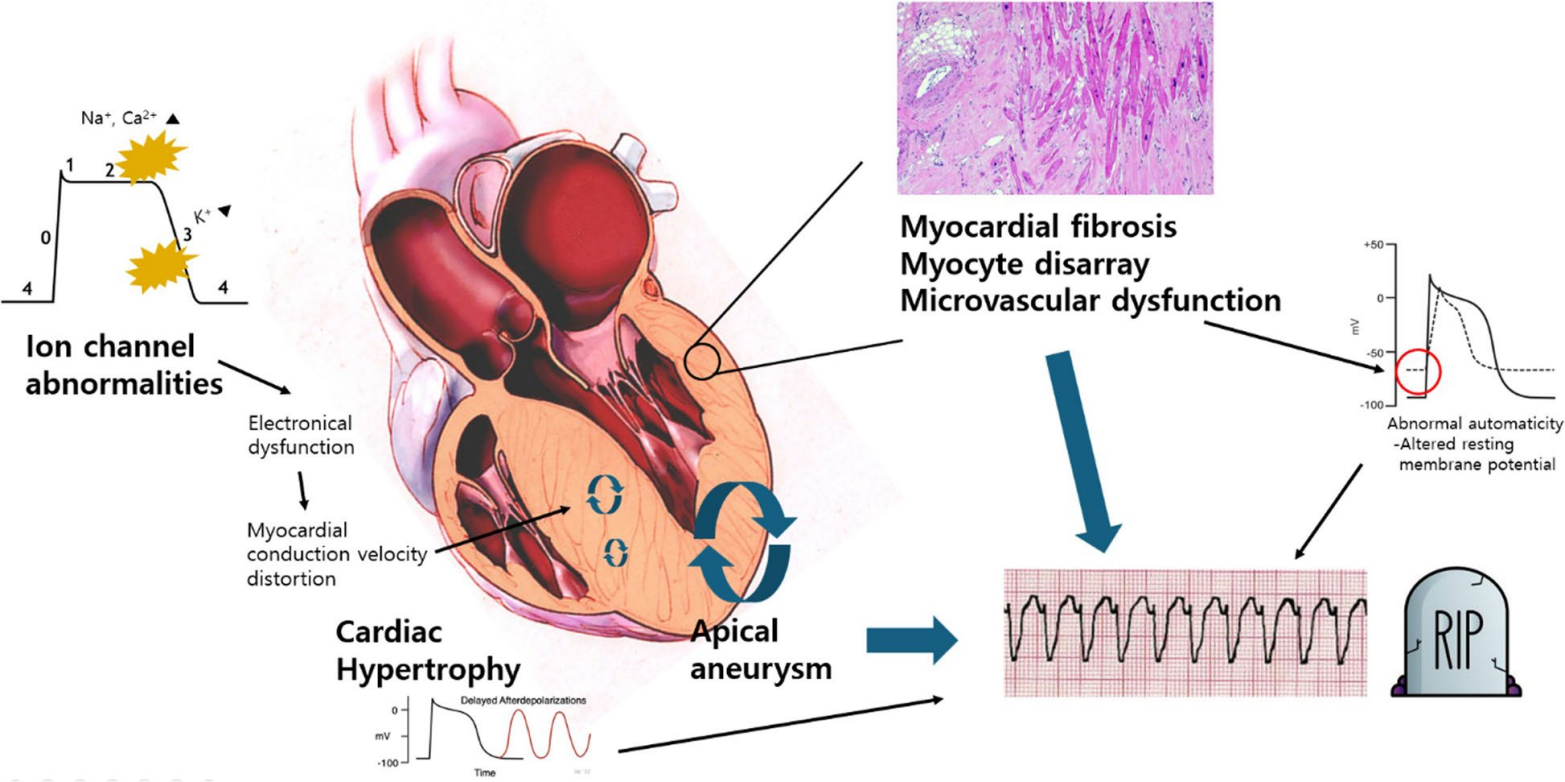
Pathophysiology of sudden cardiac death (SCD) in hypertrophic cardiomyopathy. Prevention of SCD in hypertrophic cardiomyopathy primarily involves general measures, including recommendations on physical activity, and thorough risk factor evaluations. Selecting appropriate candidates for implantable cardioverter-defibrillator implantation requires careful assessment of various risk factors and, when indicated, the 5-year SCD risk estimation score. Additionally, clinicians must understand potential complications associated with implantable cardioverter-defibrillator implantation and incorporate these considerations into clinical decisions

Two mechanisms are proposed for pathogenesis of arrhythmic SCD in the treatment of HCM: proarrhythmic structural remodeling and ion channel abnormalities (Fig. 1). Structural remodeling in the form of cardiac hypertrophy, microvascular dysfunction, myocardial fibrosis, myocyte disarray, and apical aneurysm in HCM can also serve as a proarrhythmic substrate [14, 15]. In terms of cardiac hypertrophy, increased delayed after-depolarizations (DADs) occur due to elevated cytosolic Ca^2+^ due to enhanced Ca^2+^ entry through L-type calcium channels and decreased exchange through Na^+^/Ca^2+^ exchanger routes. This increase in DADs has been proposed as a mechanism for cardiac arrhythmias associated with hypertrophy in HCM [16, 17].

Microvascular dysfunction, frequently observed in HCM, is primarily driven by reduced arteriolar density, fibrosis, myocyte disarray, and elevated left ventricle (LV) end-diastolic pressure [18–21]. Structural abnormalities of small vessels in HCM have also been demonstrated [19]. These mechanisms, combined with inadequate myocardial blood flow reserve, predispose patients to myocardial ischemia. Ischemia then promotes abnormal automaticity by altering the resting membrane potential of the myocytes, lowering the threshold for depolarization and facilitating ventricular tachycardia (VT).

Myocardial fibrosis in HCM can be visualized by cardiac magnetic resonance (CMR) imaging using late gadolinium enhancement (LGE). Myocardial scar deposition can be a common reentry circuit for arrhythmic SCD events in HCM. Previous studies have revealed a significant relationship between LGE and VT on ambulatory monitoring [22, 23]. Additionally, a greater extent of myocardial fibrosis indicated by LGE leads to an increased risk of arrhythmic SCD events [24, 25].

Myocyte disarray, which has also been identified as a risk factor for ventricular arrhythmias in HCM, can be assessed by diffusion tensor imaging. Experimentally, myocardial disarray has been linked to altered transmural distribution of connexin 43, playing the role of a substrate for cardiac arrhythmias in HCM [26, 27].

LV apical aneurysm represents another important substrate for monomorphic VT. Rowin et al. [15] reported that the annual appropriate ICD therapy rate for primary prevention in patients with apical aneurysm was 4.0%, which is approximately five times higher than in those without apical aneurysm. Given the higher prevalence of apical-dominant HCM in Asian populations, understanding the characteristics of this phenomenon is critical [28–30]. The junction between scar formation at the aneurysm rim and the adjacent myocardium consistently gives rise to monomorphic VT, providing a rationale for catheter-based ablation therapy in patients with refractory VT [15, 31].

In terms of ion channel abnormalities, lethal arrhythmias can occur, even in the earlier stages of the disease, when structural remodeling is considerably less evident [32]. Preclinical in vivo and in vitro investigations of sarcomeric mutations have revealed a spectrum of ion channel derangements [33–37]. Intracellular mechanisms, such as pathological changes in ion currents and intracellular Ca^2+^ homeostasis, play a role [38–41]. A few studies have found that altered intracellular Ca^2+^ homeostasis and increased late Na^+^ currents lead to an increased likelihood of early after-depolarizations and DADs, which contribute to arrhythmic events in diseased cardiomyocytes [17]. Recognizing this aspect, ranolazine, a potent and selective inhibitor of the cardiac late Na^+^ current, was administered to patients in the RESTYLE-HCM randomized controlled trial. The ranolazine group experienced a reduction in the 24-h burden of premature ventricular complexes, but no significant effects were seen on exercise performance, N-terminal prohormone of brain natriuretic peptide (NT-proBNP) levels, diastolic function, or quality of life [42]. As the authors themselves noted, given the study’s small sample size and the weak association between ventricular contractions and hard clinical end points, these findings should be regarded as exploratory and hypothesis generating. Their results cast doubt on the pathogenic role of this current in HCM and warrant further investigation.

### General management

Various pharmacological strategies, including the administration of prophylactic β-blockers or rhythm-modulating agents such as amiodarone, were once used to reduce the risk of SCD in young asymptomatic HCM patients [9]. These strategies are now considered part of the ICD era, with insufficient evidence to support their routine use [43].

Traditionally, patients with HCM have been advised to restrict exercise and avoid competitive sports. Recommendations are evolving, weighing the beneficial effects of mild to moderate physical activity in HCM patients, based on data from RESET-HCM clinical trial [44]. Although data addressing vigorous physical activity–related SCD are scarce, several studies have determined that vigorous physical activity is not associated with increased mortality or SCD events and may even reduce all-cause and cardiovascular mortality [45–48]. Lee et al. [49] reported that high-intensity physical activity–related SCD events were more common among younger patients, highlighting the importance of an individualized approach when prescribing exercise for HCM patients.

Prevention of SCD in HCM primarily involves general measures, including recommendations on physical activity and thorough evaluations of risk factors. Selecting appropriate candidates for ICD requires careful assessment of various risks and, when indicated, the 5-year SCD risk estimation score. Additionally, clinicians must fully understand the potential complications associated with ICDs and incorporate these considerations into clinical decision-making.

### SCD risk stratification

**Fig. 2:**
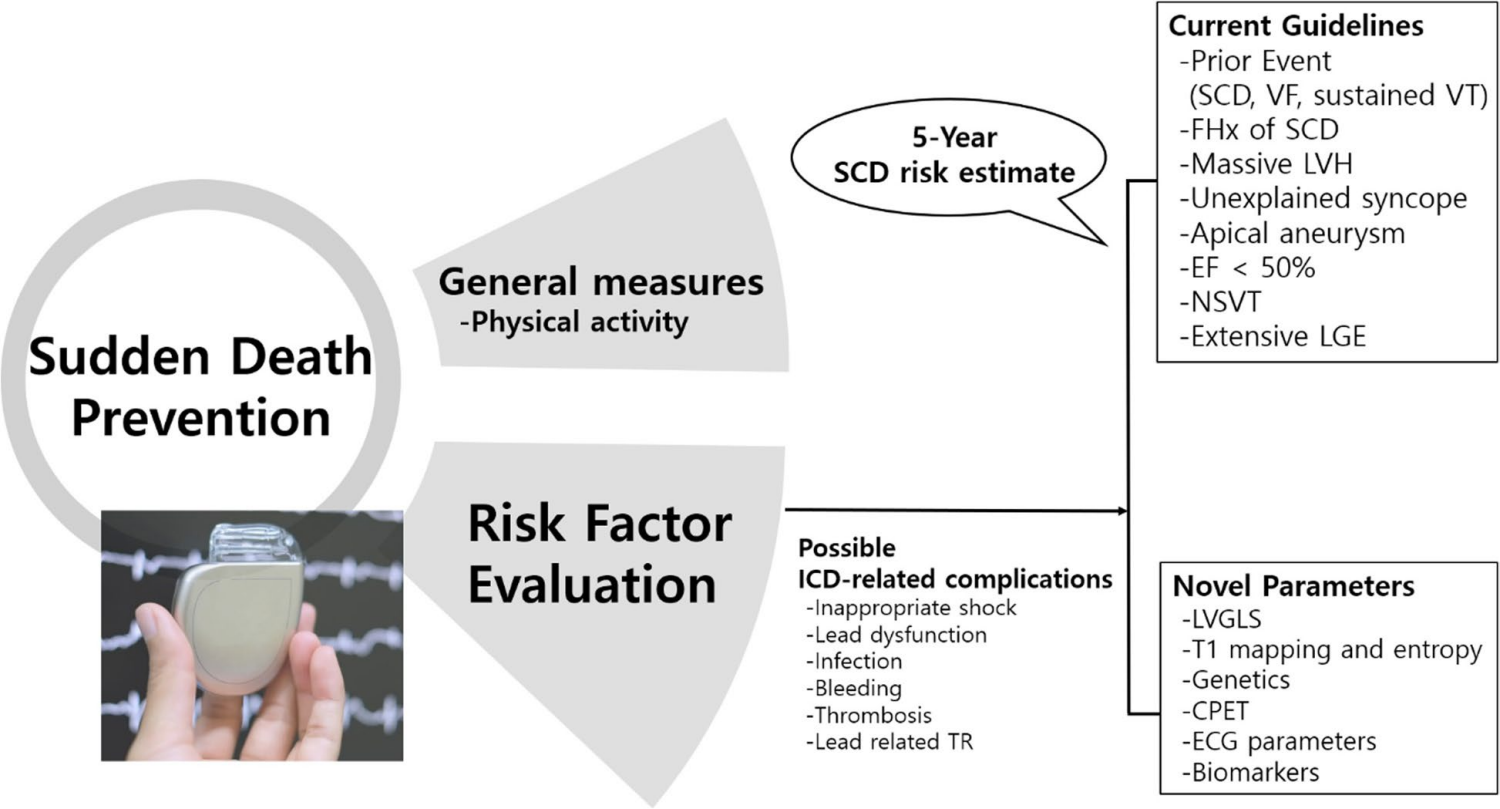
Management for prevention of sudden cardiac death (SCD) in hypertrophic cardiomyopathy. Prevention of SCD in hypertrophic cardiomyopathy primarily involves general measures, including recommendations on physical activity, and thorough risk factor evaluation. Selecting appropriate candidates for implantable cardioverter-defibrillator (ICD) implantation requires careful assessment of various risk factors and, when indicated, the 5-year SCD risk estimation score. Additionally, clinicians must fully understand potential complications associated with ICD implantation and incorporate these considerations into clinical decisions. CPET, cardiopulmonary exercise testing; ECG, electrocardiography; EF, ejection fraction; LGE, late gadolinium enhancement; LVGLS, left ventricular global longitudinal strain; LVH, left ventricular hypertrophy; NSVT, nonsustained ventricular tachycardia; TR, tricuspid regurgitation; VF, ventricular fibrillation; VT, ventricular tachycardia

Numerous studies have provided evidence supporting the significant role of ICD in preventing SCD in HCM patients. However, ICD implantation can lead to complications. For example, patients can experience inappropriate shocks, lead dysfunction, infections, bleeding, thrombosis, and lead-related tricuspid regurgitation [50]. The patient-selection criteria are important at this point, requiring consideration of general management strategies, ICD-related complications, and risk factors for SCD (Fig. 2).

Two major guidelines currently address ICD implantation in HCM patients: The 2024 guidelines from the American College of Cardiology (ACC) and American Heart Association (AHA) [51] and the 2023 European Society of Cardiology (ESC) guidelines [52]. Both guidelines strongly recommend that ICDs be considered for patients with documented cardiac arrest or hemodynamically significant ventricular arrhythmias, giving a class I indication for secondary prevention [53–57]. However, guidelines differ slightly regarding recommendations for primary prevention.

Historically, five major risk factors are considered when evaluating the risk of SCD in HCM patients: a family history of SCD, unexplained syncope, maximal LV wall thickness, nonsustained VT (NSVT) on ambulatory monitoring, and abnormal blood pressure during exercise tests. Echocardiography remains the primary imaging modality used to evaluate SCD risk in HCM, although it can underestimate maximal LV wall thickness and miss apical aneurysms [15, 58–61]. Development of new technologies, CMR imaging in particular, has offered diagnostic options to identify these risk factors. CMR can not only help quantify cardiac fibrosis by LGE or T1 mapping values, but it can also help physicians distinguish end stage (ES) HCM from other types of cardiomyopathies through echocardiography [62]. These advances, coupled with a lack of multivariate analyses demonstrating an association between abnormal blood pressure response and SCD, have led to the removal of abnormal blood pressure response from routine risk evaluation [63, 64].

**Fig. 3:**
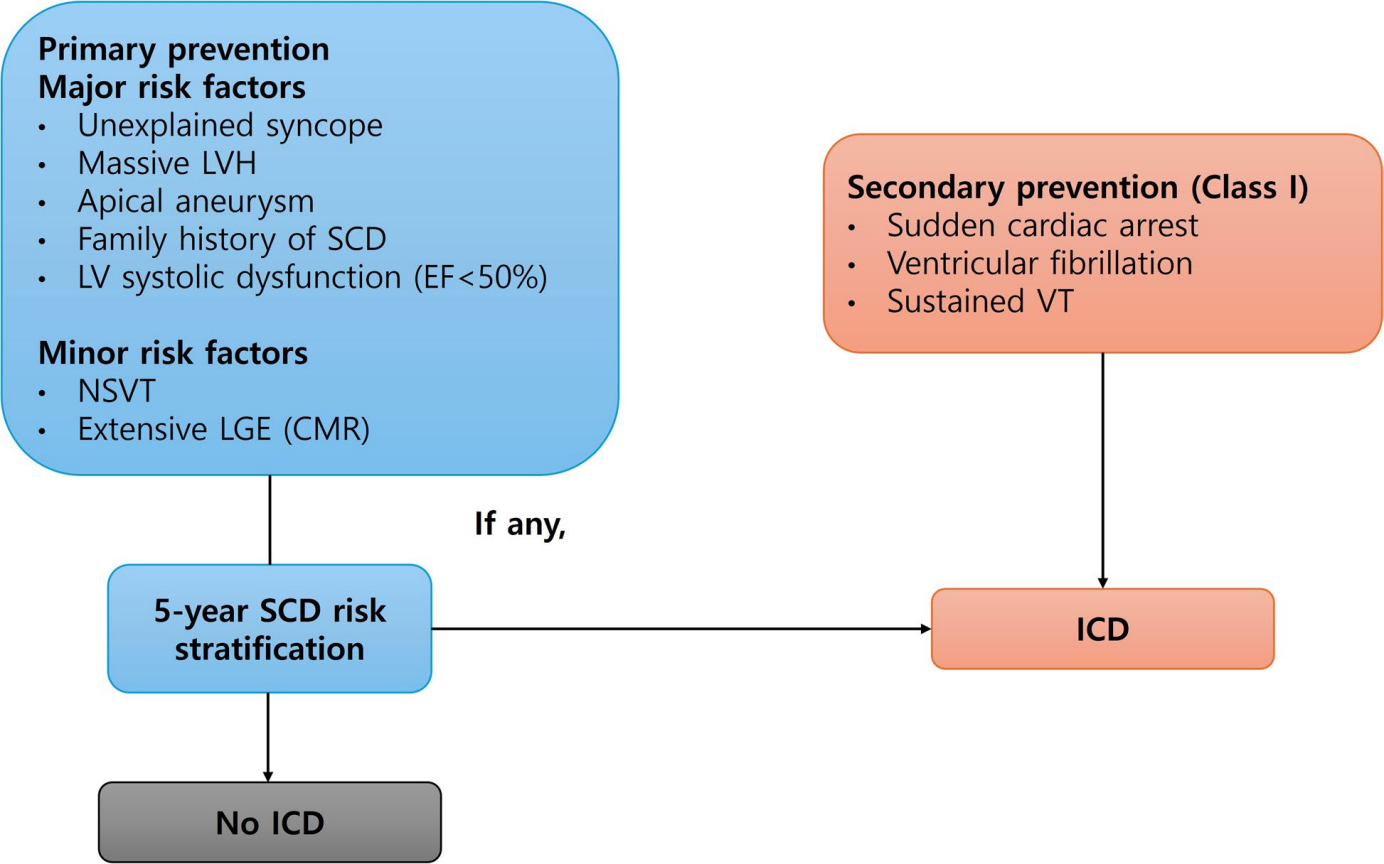
Proposed algorithm for implantable cardioverter-defibrillator (ICD) implantation in hypertrophic cardiomyopathy. Both the American College of Cardiology/American Heart Association guidelines and the European Society of Cardiology guidelines suggest class 1 indication for ICD implantation in patients who have experienced aborted sudden cardiac death (SCD), ventricular tachycardia (VT), or ventricular fibrillation (VF). In cases of one or more risk factor for SCD, the 5-year SCD risk estimate can be considered when deciding whether to install an ICD. CMR, cardiac magnetic resonance imaging; EF, ejection fraction; FH, family history; LGE, late gadolinium enhancement; LVH, left ventricular hypertrophy; NSVT, non-sustained ventricular tachycardia.

**Fig. 4:**
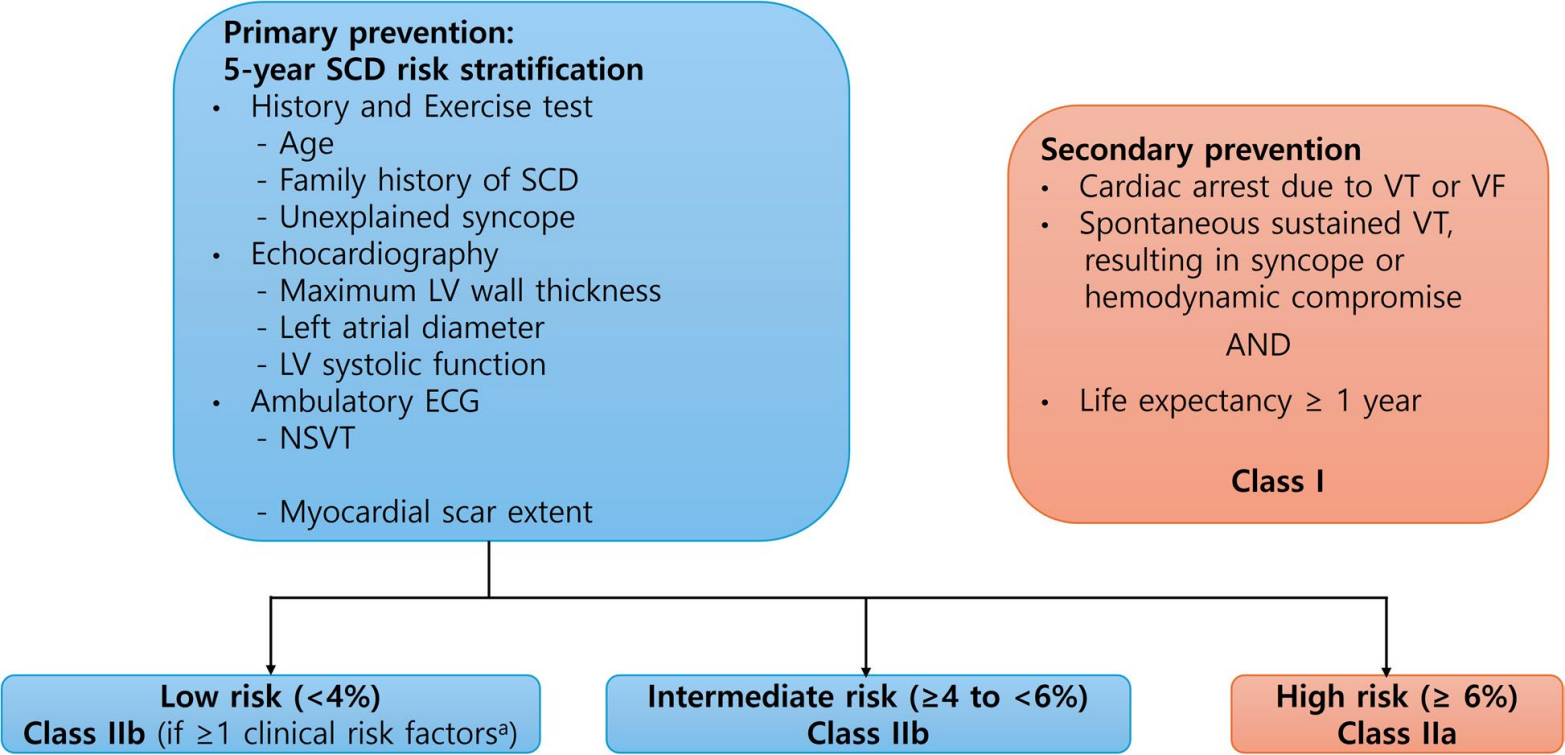
Flowchart for implantation of a cardioverter defibrillator in patients with hypertrophic cardiomyopathy (HCM). 2D, two-dimensional; ECG, electrocardiogram; ICD, implantable cardioverter defibrillator; LV, left ventricular; NSVT, nonsustained ventricular tachycardia; SCD, sudden cardiac death; VF, ventricular fibrillation; VT, ventricular tachycardia. ^a)^Clinical risk factors: extensive late gadolinium enhancement (> 15%) on cardiac magnetic resonance or LV ejection fraction < 50%.

Current ACC/AHA guidelines recommend that, for patients with one or more major risk factors, it is reasonable to use an estimate of the 5-year SCD risk to understand the magnitude of the individual risk associated with ICD decisions (Fig. 3) [11, 51]. The 5-year SCD risk score, which is well described in the ESC guidelines, is based on nine factors: age, unexplained syncope, LV outflow gradient, maximum LV wall thickness, left atrial diameter, NSVT, family history of SCD, LV systolic function, and extent of myocardial scarring. An estimated 5-year risk less than 4% is regarded as low, while that of 6% or higher implies a high risk (Fig. 4) [52]. These guidelines share a class I indication for secondary prevention in those who have suffered from aborted SCD, VT, or ventricular fibrillation (VF). In addition, because the risk of SCD extends over many decades of life, periodic reevaluations of SCD risks every 1 to 2 years are recommended [51, 65, 66]. However, given the low incidence of SCD in patients older than 60 years, this approach is more suitable for young and middle-aged individuals.

These risk stratification strategies have been validated by multiple studies in Korea. Lee et al. [67] evaluated the performance of 2020 ACC/AHA guidelines and 2014 ESC guidelines for predicting SCD in HCM. Among 1,416 HCM patients, SCD risk was elevated in those with multiple risk factors but not in those with a single risk factor. The AHA/ACC and ESC guidelines had similar performance, with the 5-year time-dependent areas under the curve showing modest statistical power (0.677 and 0.724, respectively; P = 0.235). The ESC guidelines published in 2014 were validated by Choi et al. [68], who reported high negative predictive values and accuracy for predicting SCD or appropriate ICD therapy. However, as diagnostic techniques evolve and new parameters are introduced, further validation in the Korean population will be necessary.

### Family history of sudden death from HCM

The effect of family history on SCD is based on the genetic nature of the disease. Relatives who exhibit the condition have the same genetic defect and, to some extent, share environmental factors. Several studies have examined the effects of family history as a predictor of SCD using survival analysis. Four of these studies, although they used different definitions of family history of SCD, found significant associations [69–72]. The average hazard ratio of family history of SCD (irrespective of definition) was 1.27 (95% confidence interval, 1.16–1.38) [10].

Definitions of family history of SCD continue to vary across guidelines. According to the 2023 ESC guidelines, family history is significant if at least one first-degree relative died suddenly before the age of 40 years with or without a diagnosis of HCM or when SCD occurred in a first-degree relative at any age with an established diagnosis of HCM [52]. In contrast, the 2024 AHA/ACC guidelines define a family history of SCD as a sudden death definitely or likely attributable to HCM in one or more first-degree or close relative aged 50 years or younger [51]. Close relatives would generally be second-degree relatives; however, multiple SCDs in tertiary relatives should also be considered. Multiple cases of SCD in a family history appeared to be a powerful risk factor (*P* < 0.0001), predicting frequent SCDs in childhood and adolescence [73].

### Massive left ventricular hypertrophy

LV hypertrophy (LVH) is associated with an increased prevalence of NSVT and exercise-induced ventricular arrhythmias [72, 74–76]. In HCM, both the severity and extent of LVH assessed by transthoracic echocardiogram are associated with risk of SCD [52, 74, 77, 78]. A maximum wall thickness of ≥ 30 mm in any segment within the chamber is generally regarded as the greatest risk for SCD in HCM patients, although, based on clinical judgment, a threshold of 28 mm may be a borderline value [11, 77, 79].

However, measurements from a transthoracic echocardiogram can be affected by observer variability and suboptimal imaging quality. Additionally, measuring only the maximum wall thickness may not adequately represent the full extent of myocardial hypertrophy. Under these circumstances, CMR serves as a robust additional diagnostic tool for comprehensive evaluation of LVH.

### Unexplained syncope

Spirito et al. [80] conducted a systematic investigation of the prognostic significance of syncope in more than 1,500 HCM patients in 2009. Approximately 15% of the enrolled patients had a history of syncope, either neurally mediated (vasovagal) or unexplained. Neurally mediated syncope was not associated with an increased risk of SCD. In contrast, unexplained syncope showed a relative risk of 1.78 (P = 0.08), which indicates borderline significance. Nevertheless, the authors considered this association clinically significant, given the potential mechanisms underlying syncope in HCM.

Patients with recent unexplained syncope within 6 months before initial evaluation had a fivefold higher relative risk of SCD compared with patients without syncope, regardless of age. Remote episodes of syncope (> 5 years before initial evaluation) showed no significant association in older patients.

Based on these results, current guidelines recommend treating one or more episodes of unexplained syncope, involving acute transient loss of consciousness and judged by history as unlikely to be related with neurally mediated syncope or left ventricular outflow tract obstruction as a major risk factor for SCD, particularly when occurring within 6 months of evaluation [51, 52].

### LV apical aneurysm

LV apical aneurysms are defined as a discrete, thin-walled dyskinetic or akinetic segments at the most distal portion of the LV and are characterized by transmural scarring or LGE [15, 58]. The first descriptions of LV apical aneurysms in HCM suggested an association with sustained monomorphic VT, a relatively rare event in HCM [81]. Multiple studies have demonstrated that LV apical aneurysms are a significant marker of increased SCD risk [15, 82–84]. Based on these data, the updated 2024 ACC/AHA guidelines include an apical aneurysm as one of the major risk factors for SCD [51]. In contrast, the 2023 ESC guidelines for cardiomyopathy suggest that individualized ICD decisions be based on the 5-year SCD risk score (HCM Risk-SCD score), rather than solely on the presence of an apical aneurysm [52].

### HCM with LV systolic dysfunction

HCM with LV systolic dysfunction, also known as the ES of HCM, is characterized by an ejection fraction (EF) < 50%, often accompanied by LV remodeling due to diffuse myocardial scarring [85, 86]. Numerous studies have highlighted the elevated risk of arrhythmic SCD in ES-HCM, raising concerns about the use of ICDs for primary prevention [53, 85–87]. According to the data from the Sarcomeric Human Cardiomyopathy Registry, HCM-LVSD affects about 8% of patients with HCM. Despite various natural history of HCM-LVSD, 75% of patients experienced adverse outcomes, including 35% experiencing a death equivalent (death, heart transplantation, or left ventricular assist device implantation) after a median time of 8.4 years since the development of systolic dysfunction. [88].

With advancements and aggressive implementation of various therapeutic approaches, including early evaluation for heart transplantation, cardiac resynchronization therapy, or ventricular assist devices, the annual mortality rate associated with ES-HCM has decreased from the previously reported 8% to 2%, about one-quarter of the original rate. However, according to Rowin et al. [89], ES-HCM still carries tenfold greater risk of mortality compared with HCM with preserved EF. Additionally, arrhythmic sudden death events (including appropriate ICD therapy for ventricular tachyarrhythmias, resuscitated cardiac arrest, and sudden death) were five times more frequent in ES-HCM (2.4% per Year vs. 0.5% per year, *P* = 0.006).

### NSVT on ambulatory monitor

NSVT is defined as three or more consecutive ventricular beats at a minimum rate of 120 beats per minute, lasting less than 30 s, and not resulting in hemodynamic instability. NSVT is commonly documented in approximately 20% to 35% of HCM patients, usually with 24 to 48 h of ambulatory electrocardiogram monitoring [90]. Asymptomatic NSVT has been recognized as an SCD risk factor in HCM for nearly 40 years. However, discussions continue about the reliability of NSVT as a robust marker for individual risk assessment and selecting patients for ICDs for primary prevention.

NSVT becomes more frequent as cardiac hypertrophy progresses, which likely indicates a higher degree of fibrosis and myofibrillar disarray, both of which are important predictors of the disease’s inherent arrhythmic risk [91]. Greater significance is attributed to NSVT that occurs repeatedly (three or more episodes), lasts longer (at least 10 beats), and is faster (200 beats per minute or higher), as observed over a 24- to 48-h extended ambulatory electrocardiography (ECG) monitoring period [51, 52]. Meanwhile, the value of short, single bursts of NSVT in predicting ICD-treated VT or VF remains uncertain without the presence of additional major risk factors [90].

### Extensive LGE on CMR imaging

The introduction of LGE has improved risk stratification of SCD in HCM. While LGE is widely recognized as an prognostic marker across all cardiomyopathies, the accompanying myocardial fibrosis related with CMR findings is considered arrhythmogenic in HCM [23]. As expected, a greater extent of LGE in HCM is linked to a higher burden of NSVT and an increased risk of SCD.

Current guidelines define extensive LGE as comprising ≥ 15% of LV mass, either quantified or estimated by visual inspection, based on evidence indicating a doubled risk of SCD compared with patients injected with smaller amounts of LGE [24, 51]. The ESC guidelines suggest using extensive LGE (≥ 15%) in low- to intermediate-risk category patients, helping clinicians decide on use of prophylactic ICD implantation [52]. However, quantifying LGE in HCM can be challenging due to various scarring patterns and image quality. The standard deviation (SD) threshold method, which is typically preferred in HCM, defines LGE using a specific number of SDs above a normal reference region.

A recent meta-analysis study by Kiaos et al. [92] evaluated a single study of 5,550 patients with a median follow-up of 5.2 years. When the more extensively studied 6-SD technique is used, LGE greater than 10% was the optimal cutoff and could effectively reclassify intermediate-risk patients [92]. Still, given the complexity of arrhythmogenic mechanisms in HCM, the amount of LGE is not only the problem. The pattern and location may also play a role, and further studies are warranted.

Beyond these commonly cited risk factors, studies and results on novel parameters designed to more accurately predict SCD risk have emerged.

## New perspectives on SCD risk assessment

### Left ventricular global longitudinal strain

LV global longitudinal strain (LVGLS) is more sensitive than left ventricular EF in detecting LV systolic dysfunction, showing impairments in early stages of the disease or HCM with preserved EF. Studies have shown that impaired LVGLS is associated with a significantly increased risk of SCD events and is an independent predictor of appropriate ICD therapy [93–95]. Additionally, recent studies have incorporated machine learning into LVGLS analysis in an attempt to further refine ventricular arrhythmic risk subgroups [96].

### T1 mapping and entropy on CMR in the evaluation of fibrosis

Entropy, a typical measure of image complexity, can be used to quantify tissue heterogeneity by analyzing all signal intensity values within the images. Not only LGE in CMR, but also extracellular volume fraction (ECV) and LV mean entropy derived from native T1 mapping can aid in detection of diffuse myocardial fibrosis and are independent predictors of SCD and cardiovascular disease in HCM patients [97–101]. However, T1/ECV mapping and entropy measurement have major differences. Conventional native T1 and ECV mapping derive mean values either from selected myocardial region of interests or by applying signal intensity thresholds. Even when averaged globally, both approaches may obscure regional heterogeneity. However, because entropy analysis incorporates the full distribution of voxel intensities across the entire LV, further studies are needed to determine whether entropy measurement adds incremental value over traditional region of interest or globally averaged T1/ECV metrics in detecting diffuse myocardial fibrosis in HCM.

## Genetics

Like other types of cardiomyopathies, genetic testing has been widely used in HCM. Although numerous gene mutations have been identified in HCM, the most frequently reported variants are those in the genes that express myosin binding protein C (*MYBPC3*), β-myosin heavy chain (*MYH7*), and cardiac troponin T (*TNNT2*). Among these, *TNNT2* gene mutations, which affect thin myofilament proteins, are associated with less severe hypertrophy but a higher risk of LV systolic dysfunction. By inducing severe myocyte disarray, these mutations can lead to a high incidence of SCD in younger patients [102–104].

According to a meta-analysis of 7,675 HCM patients, mutation-positive patients exhibited a higher risk of SCD by 5% (*MYPBPC3*), 11% (*MYH7*), and 17% (*TNNT2*), compared with a 0.4% risk in mutation-negative patients [105]. However, current guidelines suggest using genetic testing only for screening of HCM, not for risk stratification of SCD in HCM. In clinical practice, decisions for prophylactic ICD should not be based on genetics [51, 52].

### Cardiopulmonary exercise testing

Magrì et al. [105, 106] conducted a prospective study about the use of cardiopulmonary exercise tests in improving contemporary strategies for SCD risk stratification. The study composite end point was SCD, aborted SCD, and appropriate ICD interventions. Multivariable analysis confirmed that the exercise ventilation (VE) to CO_2_ output (VCO_2_) relationship (VE/VCO_2_ slope) was clinically significant. A VE/VCO_2_ slope cutoff value of 31 showed the highest accuracy in predicting the SCD end point within the entire HCM cohort. However, given the need for standardized protocols for cardiopulmonary exercise tests, more studies should be conducted.

### ECG parameters

Various ECG parameters can serve as predictive markers for SCD in HCM. T wave amplitude, myocardial infarction pattern (pseudo-ST segment elevation, QRS duration ≥ 120 ms, low QRS voltage), and both QRS fragmentation in ≥ 3 territories and a heart rate–corrected QT duration ≥ 460 ms were associated with ventricular arrhythmias and SCD in HCM patients [107–109]. Ventricular repolarization parameters including interval between the peak and end of the electrocardiographic T wave (Tpe) to corrected QT interval ratio and Tpe interval were also related to a higher risk of VT [110, 111].

### Biomarkers

An increased level of NT-proBNP was an independent predictor of SCD in patients with HCM [112]. It was also significantly correlated with cardiac fibrosis, as detected by either LGE or Masson’s trichrome staining in the myocardium.

## Conclusions

With advances in our understanding of HCM using diagnostic modalities beyond echocardiography, updates have been incorporated into both ACC/AHA and ESC guidelines. Moreover, as genetics advances and artificial intelligence evolves, clinical studies are focusing on improving risk stratification. However, clinicians must not depend solely on single aspects of the disease. Instead, they must employ multiple tools to evaluate the risk of SCD in HCM patients. Last, as current guidelines suggest, it is important to reevaluate a patient’s heart every 1 to 2 years, even in asymptomatic cases. Although some negative findings regarding longitudinal changes have been reported, the importance of regular follow-up remains indisputable [113].

## Data Availability

No datasets were generated or analysed during the current study.

## Abbreviations

ACC: American College of Cardiology
AHA: American Heart Association
CMR: Cardiac magnetic resonance
DAD: Delayed after-depolarizations
ECG: Electrocardiography
ECV: Extracellular volume fraction
EF: Ejection fraction
ES: End stage
ESC: European Society of Cardiology
HCM: Hypertrophic cardiomyopathy
ICD: Implantable cardioverter-defibrillator
LGE: Late gadolinium enhancement
LV: Left ventricle
LVGLS: Left ventricular global longitudinal strain
LVH: Left ventricular hypertrophy
NSVT: Nonsustained ventricular tachycardia
NT-proBNP: N-terminal prohormone of brain natriuretic peptide
SCD: Sudden cardiac death
SD: Standard deviation
Tpe: Interval between the peak and end of the electrocardiographic T wave
VCO_2_: CO_2_ output
VE: Ventilation
VF: Ventricular fibrillation
VT: Ventricular tachycardia

## References

[1] Maron BJ. Clinical course and management of hypertrophic cardiomyopathy. N Engl J Med. 2018;379:655–68. 30110588 10.1056/NEJMra1710575

[2] Maron BJ, Rowin EJ, Maron MS. Global burden of hypertrophic cardiomyopathy. JACC Heart Fail. 2018;6:376–8. 29724362 10.1016/j.jchf.2018.03.004

[3] Braunwald E, Lambrew CT, Rockoff SD, Ross J, Morrow AG. Idiopathic hypertrophic subaortic stenosis: I. A description of the disease based upon an analysis of 64 patients. Circulation. 1964;30:3–119. 10.1161/01.cir.29.5s4.iv-3 14227306

[4] Maron BJ, Rowin EJ, Maron MS. Paradigm of sudden death prevention in hypertrophic cardiomyopathy. Circ Res. 2019;125:370–8. 31518168 10.1161/CIRCRESAHA.119.315159

[5] Maron BJ, Rowin EJ, Maron MS. Evolution of risk stratification and sudden death prevention in hypertrophic cardiomyopathy: twenty years with the implantable cardioverter-defibrillator. Heart Rhythm. 2021;18:1012–23. 33508516 10.1016/j.hrthm.2021.01.019

[6] Maron BJ, Rowin EJ, Casey SA, Maron MS. How hypertrophic cardiomyopathy became a contemporary treatable genetic disease with low mortality: shaped by 50 years of clinical research and practice. JAMA Cardiol. 2016;1:98–105. 27437663 10.1001/jamacardio.2015.0354

[7] Maron BJ, Maron MS, Rowin EJ. Perspectives on the overall risks of living with hypertrophic cardiomyopathy. Circulation. 2017;135:2317–9. 28606946 10.1161/CIRCULATIONAHA.117.027738

[8] Maron BJ, Maron MS. Contemporary strategies for risk stratification and prevention of sudden death with the implantable defibrillator in hypertrophic cardiomyopathy. Heart Rhythm. 2016;13:1155–65. 26749314 10.1016/j.hrthm.2015.12.048

[9] Christiaans I, van Engelen K, van Langen IM, Birnie E, Bonsel GJ, Elliott PM, et al. Risk stratification for sudden cardiac death in hypertrophic cardiomyopathy: systematic review of clinical risk markers. Europace. 2010;12:313–21. 20118111 10.1093/europace/eup431

[10] O’Mahony C, Jichi F, Pavlou M, Monserrat L, Anastasakis A, Rapezzi C, et al. A novel clinical risk prediction model for sudden cardiac death in hypertrophic cardiomyopathy (HCM risk-SCD). Eur Heart J. 2014;35:2010–20. 24126876 10.1093/eurheartj/eht439

[11] Maron BJ, Doerer JJ, Haas TS, Tierney DM, Mueller FO. Sudden deaths in young competitive athletes: analysis of 1866 deaths in the United States, 1980–2006. Circulation. 2009;119:1085–92. 19221222 10.1161/CIRCULATIONAHA.108.804617

[12] Maron BJ, Shen WK, Link MS, Epstein AE, Almquist AK, Daubert JP, et al. Efficacy of implantable cardioverter-defibrillators for the prevention of sudden death in patients with hypertrophic cardiomyopathy. N Engl J Med. 2000;342:365–73. 10666426 10.1056/NEJM200002103420601

[13] Marian AJ, Asatryan B, Wehrens XH. Genetic basis and molecular biology of cardiac arrhythmias in cardiomyopathies. Cardiovasc Res. 2020;116:1600–19. 32348453 10.1093/cvr/cvaa116 PMC7341170

[14] Rowin EJ, Maron BJ, Haas TS, Garberich RF, Wang W, Link MS, et al. Hypertrophic cardiomyopathy with left ventricular apical aneurysm: implications for risk stratification and management. J Am Coll Cardiol. 2017;69:761–73. 28209216 10.1016/j.jacc.2016.11.063

[15] Coppini R, Ferrantini C, Mugelli A, Poggesi C, Cerbai E. Altered Ca and Na homeostasis in human hypertrophic cardiomyopathy: implications for arrhythmogenesis. Front Physiol. 2018;9:1391. 30420810 10.3389/fphys.2018.01391 PMC6215954

[16] Coppini R, Santini L, Olivotto I, Ackerman MJ, Cerbai E. Abnormalities in sodium current and calcium homoeostasis as drivers of arrhythmogenesis in hypertrophic cardiomyopathy. Cardiovasc Res. 2020;116:1585–99. 32365196 10.1093/cvr/cvaa124

[17] Cecchi F, Olivotto I, Gistri R, Lorenzoni R, Chiriatti G, Camici PG. Coronary microvascular dysfunction and prognosis in hypertrophic cardiomyopathy. N Engl J Med. 2003;349:1027–35. 12968086 10.1056/NEJMoa025050

[18] Maron BJ, Wolfson JK, Epstein SE, Roberts WC. Intramural (“small vessel”) coronary artery disease in hypertrophic cardiomyopathy. J Am Coll Cardiol. 1986;8:545–57. 3745699 10.1016/s0735-1097(86)80181-4

[19] Schwartzkopff B, Mundhenke M, Strauer BE. Alterations of the architecture of subendocardial arterioles in patients with hypertrophic cardiomyopathy and impaired coronary vasodilator reserve: a possible cause for myocardial ischemia. J Am Coll Cardiol. 1998;31:1089–96. 9562012 10.1016/s0735-1097(98)00036-9

[20] Cannon RO, Rosing DR, Maron BJ, Leon MB, Bonow RO, Watson RM, et al. Myocardial ischemia in patients with hypertrophic cardiomyopathy: contribution of inadequate vasodilator reserve and elevated left ventricular filling pressures. Circulation. 1985;71:234–43. 4038383 10.1161/01.cir.71.2.234

[21] Adabag AS, Maron BJ, Appelbaum E, Harrigan CJ, Buros JL, Gibson CM, et al. Occurrence and frequency of arrhythmias in hypertrophic cardiomyopathy in relation to delayed enhancement on cardiovascular magnetic resonance. J Am Coll Cardiol. 2008;51:1369–74. 18387438 10.1016/j.jacc.2007.11.071

[22] Weissler-Snir A, Hindieh W, Spears DA, Adler A, Rakowski H, Chan RH. The relationship between the quantitative extent of late gadolinium enhancement and burden of nonsustained ventricular tachycardia in hypertrophic cardiomyopathy: a delayed contrast-enhanced magnetic resonance study. J Cardiovasc Electrophysiol. 2019;30:651–7. 30680853 10.1111/jce.13855

[23] Chan RH, Maron BJ, Olivotto I, Pencina MJ, Assenza GE, Haas T, et al. Prognostic value of quantitative contrast-enhanced cardiovascular magnetic resonance for the evaluation of sudden death risk in patients with hypertrophic cardiomyopathy. Circulation. 2014;130:484–95. 25092278 10.1161/CIRCULATIONAHA.113.007094

[24] Weng Z, Yao J, Chan RH, He J, Yang X, Zhou Y, et al. Prognostic value of LGE-CMR in HCM: a meta-analysis. JACC Cardiovasc Imaging. 2016;9:1392–402. 27450876 10.1016/j.jcmg.2016.02.031

[25] Ariga R, Tunnicliffe EM, Manohar SG, Mahmod M, Raman B, Piechnik SK, et al. Identification of myocardial disarray in patients with hypertrophic cardiomyopathy and ventricular arrhythmias. J Am Coll Cardiol. 2019;73:2493–502. 31118142 10.1016/j.jacc.2019.02.065 PMC6548973

[26] Ripplinger CM, Li W, Hadley J, Chen J, Rothenberg F, Lombardi R, et al. Enhanced transmural fiber rotation and connexin 43 heterogeneity are associated with an increased upper limit of vulnerability in a transgenic rabbit model of human hypertrophic cardiomyopathy. Circ Res. 2007;101:1049–57. 17885214 10.1161/CIRCRESAHA.107.161240 PMC2366809

[27] Moon J, Shim CY, Ha JW, Cho IJ, Kang MK, Yang WI, et al. Clinical and echocardiographic predictors of outcomes in patients with apical hypertrophic cardiomyopathy. Am J Cardiol. 2011;108:1614–9. 21890076 10.1016/j.amjcard.2011.07.024

[28] Kubo T, Kitaoka H, Okawa M, Hirota T, Hoshikawa E, Hayato K, et al. Clinical profiles of hypertrophic cardiomyopathy with apical phenotype: comparison of pure-apical form and distal-dominant form. Circ J. 2009;73:2330–6. 19838003 10.1253/circj.cj-09-0438

[29] Yin Y, Hu W, Zhang L, Wu D, Yang C, Ye X. Clinical, echocardiographic and cardiac MRI predictors of outcomes in patients with apical hypertrophic cardiomyopathy. Int J Cardiovasc Imaging. 2022;38:643–51. 34652588 10.1007/s10554-021-02430-w

[30] Lim KK, Maron BJ, Knight BP. Successful catheter ablation of hemodynamically unstable monomorphic ventricular tachycardia in a patient with hypertrophic cardiomyopathy and apical aneurysm. J Cardiovasc Electrophysiol. 2009;20:445–7. 19054248 10.1111/j.1540-8167.2008.01366.x

[31] Maurizi N, Passantino S, Spaziani G, Girolami F, Arretini A, Targetti M, et al. Long-term outcomes of pediatric-onset hypertrophic cardiomyopathy and age-specific risk factors for lethal arrhythmic events. JAMA Cardiol. 2018;3:520–5. 29710196 10.1001/jamacardio.2018.0789 PMC6128509

[32] Knollmann BC, Kirchhof P, Sirenko SG, Degen H, Greene AE, Schober T, et al. Familial hypertrophic cardiomyopathy-linked mutant troponin T causes stress-induced ventricular tachycardia and Ca2+-dependent action potential remodeling. Circ Res. 2003;92:428–36. 12600890 10.1161/01.RES.0000059562.91384.1A

[33] Fraysse B, Weinberger F, Bardswell SC, Cuello F, Vignier N, Geertz B, et al. Increased myofilament Ca2+ sensitivity and diastolic dysfunction as early consequences of Mybpc3 mutation in heterozygous knock-in mice. J Mol Cell Cardiol. 2012;52:1299–307. 22465693 10.1016/j.yjmcc.2012.03.009 PMC3370652

[34] Han L, Li Y, Tchao J, Kaplan AD, Lin B, Li Y, et al. Study familial hypertrophic cardiomyopathy using patient-specific induced pluripotent stem cells. Cardiovasc Res. 2014;104:258–69. 25209314 10.1093/cvr/cvu205 PMC4217687

[35] Helms AS, Alvarado FJ, Yob J, Tang VT, Pagani F, Russell MW, et al. Genotype-dependent and -independent calcium signaling dysregulation in human hypertrophic cardiomyopathy. Circulation. 2016;134:1738–48. 27688314 10.1161/CIRCULATIONAHA.115.020086 PMC5127749

[36] Flenner F, Jungen C, Küpker N, Ibel A, Kruse M, Koivumäki JT, et al. Translational investigation of electrophysiology in hypertrophic cardiomyopathy. J Mol Cell Cardiol. 2021;157:77–89. 33957110 10.1016/j.yjmcc.2021.04.009 PMC8320769

[37] Coppini R, Mazzoni L, Ferrantini C, Gentile F, Pioner JM, Laurino A, et al. Ranolazine prevents phenotype development in a mouse model of hypertrophic cardiomyopathy. Circ Heart Fail. 2017;10: e003565. 28255011 10.1161/CIRCHEARTFAILURE.116.003565 PMC6284403

[38] Coppini R, Ferrantini C, Yao L, Fan P, Del Lungo M, Stillitano F, et al. Late sodium current inhibition reverses electromechanical dysfunction in human hypertrophic cardiomyopathy. Circulation. 2013;127:575–84. 23271797 10.1161/CIRCULATIONAHA.112.134932

[39] Ferrantini C, Coppini R, Pioner JM, Gentile F, Tosi B, Mazzoni L, et al. Pathogenesis of hypertrophic cardiomyopathy is mutation rather than disease specific: a comparison of the cardiac troponin T E163R and R92Q mouse models. J Am Heart Assoc. 2017;6: e005407. 28735292 10.1161/JAHA.116.005407 PMC5586279

[40] Ferrantini C, Pioner JM, Mazzoni L, Gentile F, Tosi B, Rossi A, et al. Late sodium current inhibitors to treat exercise-induced obstruction in hypertrophic cardiomyopathy: an in vitro study in human myocardium. Br J Pharmacol. 2018;175:2635–52. 29579779 10.1111/bph.14223 PMC6003658

[41] Olivotto I, Camici PG, Merlini PA, Rapezzi C, Patten M, Climent V, et al. Efficacy of ranolazine in patients with symptomatic hypertrophic cardiomyopathy: the RESTYLE-HCM randomized, double-blind, placebo-controlled study. Circ Heart Fail. 2018;11: e004124. 29321131 10.1161/CIRCHEARTFAILURE.117.004124

[42] Melacini P, Maron BJ, Bobbo F, Basso C, Tokajuk B, Zucchetto M, et al. Evidence that pharmacological strategies lack efficacy for the prevention of sudden death in hypertrophic cardiomyopathy. Heart. 2007;93:708–10. 17502652 10.1136/hrt.2006.099416 PMC1955204

[43] Saberi S, Wheeler M, Bragg-Gresham J, Hornsby W, Agarwal PP, Attili A, et al. Effect of moderate-intensity exercise training on peak oxygen consumption in patients with hypertrophic cardiomyopathy: a randomized clinical trial. JAMA. 2017;317:1349–57. 28306757 10.1001/jama.2017.2503 PMC5469299

[44] Kwon S, Lee HJ, Han KD, Kim DH, Lee SP, Hwang IC, et al. Association of physical activity with all-cause and cardiovascular mortality in 7666 adults with hypertrophic cardiomyopathy (HCM): more physical activity is better. Br J Sports Med. 2021;55:1034–40. 32967852 10.1136/bjsports-2020-101987

[45] Lampert R, Ackerman MJ, Marino BS, Burg M, Ainsworth B, Salberg L, et al. Vigorous exercise in patients with hypertrophic cardiomyopathy. JAMA Cardiol. 2023;8:595–605. 37195701 10.1001/jamacardio.2023.1042 PMC10193262

[46] Martinez KA, Bos JM, Baggish AL, Phelan DM, Tobert KE, Newman DB, et al. Return-to-play for elite athletes with genetic heart diseases predisposing to sudden cardiac death. J Am Coll Cardiol. 2023;82:661–70. 37587576 10.1016/j.jacc.2023.05.059

[47] Pelliccia A, Caselli S, Pelliccia M, Musumeci MB, Lemme E, Di Paolo FM, et al. Clinical outcomes in adult athletes with hypertrophic cardiomyopathy: a 7-year follow-up study. Br J Sports Med. 2020;54:1008–12. 32532845 10.1136/bjsports-2019-100890

[48] Lee HJ, Gwak SY, Kim K, Cho I, Shim CY, Ha JW, et al. Factors associated with high-intensity physical activity and sudden cardiac death in hypertrophic cardiomyopathy. Heart. 2025;111:253–61. 39848653 10.1136/heartjnl-2024-324928

[49] Kirkfeldt RE, Johansen JB, Nohr EA, Jørgensen OD, Nielsen JC. Complications after cardiac implantable electronic device implantations: an analysis of a complete, nationwide cohort in Denmark. Eur Heart J. 2014;35:1186–94. 24347317 10.1093/eurheartj/eht511 PMC4012708

[50] Ommen SR, Ho CY, Asif IM, Balaji S, Burke MA, Day SM, et al. 2024 AHA/ACC/AMSSM/HRS/PACES/SCMR guideline for the management of hypertrophic cardiomyopathy: a report of the American Heart Association/American College of Cardiology Joint Committee on clinical practice guidelines. Circulation. 2024;149:e1239–311. 38718139 10.1161/CIR.0000000000001250

[51] Arbelo E, Protonotarios A, Gimeno JR, Arbustini E, Barriales-Villa R, Basso C, et al. 2023 ESC guidelines for the management of cardiomyopathies. Eur Heart J. 2023;44:3503–626. 37622657 10.1093/eurheartj/ehad194

[52] Maron MS, Rowin EJ, Wessler BS, Mooney PJ, Fatima A, Patel P, et al. Enhanced American College of Cardiology/American Heart Association Strategy for prevention of sudden cardiac death in high-risk patients with hypertrophic cardiomyopathy. JAMA Cardiol. 2019;4:644–57. 31116360 10.1001/jamacardio.2019.1391 PMC6537832

[53] O’Mahony C, Tome-Esteban M, Lambiase PD, Pantazis A, Dickie S, McKenna WJ, et al. A validation study of the 2003 American College of Cardiology/European Society of Cardiology and 2011 American College of Cardiology Foundation/American Heart Association risk stratification and treatment algorithms for sudden cardiac death in patients with hypertrophic cardiomyopathy. Heart. 2013;99:534–41. 23339826 10.1136/heartjnl-2012-303271

[54] Maron BJ, Spirito P, Shen WK, Haas TS, Formisano F, Link MS, et al. Implantable cardioverter-defibrillators and prevention of sudden cardiac death in hypertrophic cardiomyopathy. JAMA. 2007;298:405–12. 17652294 10.1001/jama.298.4.405

[55] Vriesendorp PA, Schinkel AF, Van Cleemput J, Willems R, Jordaens LJ, Theuns DA, et al. Implantable cardioverter-defibrillators in hypertrophic cardiomyopathy: patient outcomes, rate of appropriate and inappropriate interventions, and complications. Am Heart J. 2013;166:496–502. 24016499 10.1016/j.ahj.2013.06.009

[56] Elliott PM, Sharma S, Varnava A, Poloniecki J, Rowland E, McKenna WJ. Survival after cardiac arrest or sustained ventricular tachycardia in patients with hypertrophic cardiomyopathy. J Am Coll Cardiol. 1999;33:1596–601. 10334430 10.1016/s0735-1097(99)00056-x

[57] Ichida M, Nishimura Y, Kario K. Clinical significance of left ventricular apical aneurysms in hypertrophic cardiomyopathy patients: the role of diagnostic electrocardiography. J Cardiol. 2014;64:265–72. 24674752 10.1016/j.jjcc.2014.02.011

[58] Corona-Villalobos CP, Sorensen LL, Pozios I, Chu L, Eng J, Abraham MR, et al. Left ventricular wall thickness in patients with hypertrophic cardiomyopathy: a comparison between cardiac magnetic resonance imaging and echocardiography. Int J Cardiovasc Imaging. 2016;32:945–54. 26896038 10.1007/s10554-016-0858-4

[59] Bois JP, Geske JB, Foley TA, Ommen SR, Pellikka PA. Comparison of maximal wall thickness in hypertrophic cardiomyopathy differs between magnetic resonance imaging and transthoracic echocardiography. Am J Cardiol. 2017;119:643–50. 27956002 10.1016/j.amjcard.2016.11.010

[60] Maron MS, Lesser JR, Maron BJ. Management implications of massive left ventricular hypertrophy in hypertrophic cardiomyopathy significantly underestimated by echocardiography but identified by cardiovascular magnetic resonance. Am J Cardiol. 2010;105:1842–3. 20538141 10.1016/j.amjcard.2010.01.367

[61] Kim GH, Kim CM, Jang BH, Lee HH, Hong S, Eum SH, et al. Findings of cardiac magnetic resonance imaging in hypertrophic cardiomyopathy after 16 years. J Cardiovasc Ultrasound. 2016;24:239–42. 27721955 10.4250/jcu.2016.24.3.239 PMC5050313

[62] Monda E, Limongelli G. Integrated sudden cardiac death risk prediction model for patients with hypertrophic cardiomyopathy. Circulation. 2023;147:281–3. 36689567 10.1161/CIRCULATIONAHA.122.063019

[63] Rodrigues T, Raposo SC, Brito D, Lopes LR. Prognostic relevance of exercise testing in hypertrophic cardiomyopathy: a systematic review. Int J Cardiol. 2021;339:83–92. 34214502 10.1016/j.ijcard.2021.06.051 PMC8425182

[64] O’Mahony C, Jichi F, Ommen SR, Christiaans I, Arbustini E, Garcia-Pavia P, et al. International external validation study of the 2014 European Society of Cardiology Guidelines on sudden cardiac death prevention in hypertrophic cardiomyopathy (EVIDENCE-HCM). Circulation. 2018;137:1015–23. 29191938 10.1161/CIRCULATIONAHA.117.030437

[65] Maron BJ, Rowin EJ, Casey SA, Link MS, Lesser JR, Chan RH, et al. Hypertrophic cardiomyopathy in adulthood associated with low cardiovascular mortality with contemporary management strategies. J Am Coll Cardiol. 2015;65:1915–28. 25953744 10.1016/j.jacc.2015.02.061

[66] Lee HJ, Kim HK, Lee SC, Kim J, Park JB, Lee SP, et al. Performance of 2020 AHA/ACC HCM guidelines and incremental value of myocardial strain for predicting SCD. JACC Asia. 2024;4:10–22. 38222259 10.1016/j.jacasi.2023.09.002 PMC10782402

[67] Choi YJ, Kim HK, Lee SC, Park JB, Moon I, Park J, et al. Validation of the hypertrophic cardiomyopathy risk-sudden cardiac death calculator in Asians. Heart. 2019;105:1892–7. 31383719 10.1136/heartjnl-2019-315160

[68] Elliott PM, Gimeno JR, Tomé MT, Shah J, Ward D, Thaman R, et al. Left ventricular outflow tract obstruction and sudden death risk in patients with hypertrophic cardiomyopathy. Eur Heart J. 2006;27:1933–41. 16754630 10.1093/eurheartj/ehl041

[69] Maki S, Ikeda H, Muro A, Yoshida N, Shibata A, Koga Y, et al. Predictors of sudden cardiac death in hypertrophic cardiomyopathy. Am J Cardiol. 1998;82:774–8. 9761089 10.1016/s0002-9149(98)00455-x

[70] D’Andrea A, Caso P, Severino S, Cuomo S, Capozzi G, Calabrò P, et al. Prognostic value of intra-left ventricular electromechanical asynchrony in patients with hypertrophic cardiomyopathy. Eur Heart J. 2006;27:1311–8. 16364972 10.1093/eurheartj/ehi688

[71] Gimeno JR, Tomé-Esteban M, Lofiego C, Hurtado J, Pantazis A, Mist B, et al. Exercise-induced ventricular arrhythmias and risk of sudden cardiac death in patients with hypertrophic cardiomyopathy. Eur Heart J. 2009;30:2599–605. 19689975 10.1093/eurheartj/ehp327

[72] Dimitrow PP, Chojnowska L, Rudzinski T, Piotrowski W, Ziólkowska L, Wojtarowicz A, et al. Sudden death in hypertrophic cardiomyopathy: old risk factors re-assessed in a new model of maximalized follow-up. Eur Heart J. 2010;31:3084–93. 20843960 10.1093/eurheartj/ehq308

[73] Elliott PM, Gimeno Blanes JR, Mahon NG, Poloniecki JD, McKenna WJ. Relation between severity of left-ventricular hypertrophy and prognosis in patients with hypertrophic cardiomyopathy. Lancet. 2001;357:420–4. 11273061 10.1016/S0140-6736(00)04005-8

[74] Adabag AS, Casey SA, Kuskowski MA, Zenovich AG, Maron BJ. Spectrum and prognostic significance of arrhythmias on ambulatory Holter electrocardiogram in hypertrophic cardiomyopathy. J Am Coll Cardiol. 2005;45:697–704. 15734613 10.1016/j.jacc.2004.11.043

[75] Spirito P, Watson RM, Maron BJ. Relation between extent of left ventricular hypertrophy and occurrence of ventricular tachycardia in hypertrophic cardiomyopathy. Am J Cardiol. 1987;60:1137–42. 2961234 10.1016/0002-9149(87)90406-1

[76] Spirito P, Bellone P, Harris KM, Bernabo P, Bruzzi P, Maron BJ. Magnitude of left ventricular hypertrophy and risk of sudden death in hypertrophic cardiomyopathy. N Engl J Med. 2000;342:1778–85. 10853000 10.1056/NEJM200006153422403

[77] Jensen MK, Jacobsson L, Almaas V, van Buuren F, Hansen PR, Hansen TF, et al. Influence of septal thickness on the clinical outcome after alcohol septal alation in hypertrophic cardiomyopathy. Circ Cardiovasc Interv. 2016;9: e003214. 27217377 10.1161/CIRCINTERVENTIONS.115.003214

[78] Monserrat L, Elliott PM, Gimeno JR, Sharma S, Penas-Lado M, McKenna WJ. Non-sustained ventricular tachycardia in hypertrophic cardiomyopathy: an independent marker of sudden death risk in young patients. J Am Coll Cardiol. 2003;42:873–9. 12957435 10.1016/s0735-1097(03)00827-1

[79] Spirito P, Autore C, Rapezzi C, Bernabò P, Badagliacca R, Maron MS, et al. Syncope and risk of sudden death in hypertrophic cardiomyopathy. Circulation. 2009;119:1703–10. 19307481 10.1161/CIRCULATIONAHA.108.798314

[80] Alfonso F, Frenneaux MP, McKenna WJ. Clinical sustained uniform ventricular tachycardia in hypertrophic cardiomyopathy: association with left ventricular apical aneurysm. Br Heart J. 1989;61:178–81. 2923756 10.1136/hrt.61.2.178 PMC1216637

[81] Papanastasiou CA, Zegkos T, Karamitsos TD, Rowin EJ, Maron MS, Parcharidou D, et al. Prognostic role of left ventricular apical aneurysm in hypertrophic cardiomyopathy: a systematic review and meta-analysis. Int J Cardiol. 2021;332:127–32. 33794232 10.1016/j.ijcard.2021.03.056

[82] Maron MS, Finley JJ, Bos JM, Hauser TH, Manning WJ, Haas TS, et al. Prevalence, clinical significance, and natural history of left ventricular apical aneurysms in hypertrophic cardiomyopathy. Circulation. 2008;118:1541–9. 18809796 10.1161/CIRCULATIONAHA.108.781401

[83] Minami Y, Haruki S, Hagiwara N. Phenotypic overlap in hypertrophic cardiomyopathy: apical hypertrophy, midventricular obstruction, and apical aneurysm. J Cardiol. 2014;64:463–9. 24768408 10.1016/j.jjcc.2014.03.003

[84] Harris KM, Spirito P, Maron MS, Zenovich AG, Formisano F, Lesser JR, et al. Prevalence, clinical profile, and significance of left ventricular remodeling in the end-stage phase of hypertrophic cardiomyopathy. Circulation. 2006;114:216–25. 16831987 10.1161/CIRCULATIONAHA.105.583500

[85] Kawarai H, Kajimoto K, Minami Y, Hagiwara N, Kasanuki H. Risk of sudden death in end-stage hypertrophic cardiomyopathy. J Card Fail. 2011;17:459–64. 21624733 10.1016/j.cardfail.2011.01.015

[86] Biagini E, Coccolo F, Ferlito M, Perugini E, Rocchi G, Bacchi-Reggiani L, et al. Dilated-hypokinetic evolution of hypertrophic cardiomyopathy: prevalence, incidence, risk factors, and prognostic implications in pediatric and adult patients. J Am Coll Cardiol. 2005;46:1543–50. 16226182 10.1016/j.jacc.2005.04.062

[87] Marstrand P, Han L, Day SM, Olivotto I, Ashley EA, Michels M, et al. Hypertrophic cardiomyopathy with left ventricular systolic dysfunction: insights from the SHaRe registry. Circulation. 2020;141:1371–83. 32228044 10.1161/CIRCULATIONAHA.119.044366 PMC7182243

[88] Rowin EJ, Maron BJ, Carrick RT, Patel PP, Koethe B, Wells S, et al. Outcomes in patients with hypertrophic cardiomyopathy and left ventricular systolic dysfunction. J Am Coll Cardiol. 2020;75:3033–43. 32553256 10.1016/j.jacc.2020.04.045

[89] Wang W, Lian Z, Rowin EJ, Maron BJ, Maron MS, Link MS. Prognostic implications of nonsustained ventricular tachycardia in high-risk patients with hypertrophic cardiomyopathy. Circ Arrhythm Electrophysiol. 2017;10: e004604. 28314849 10.1161/CIRCEP.116.004604

[90] Spirito P, Rapezzi C, Autore C, Bruzzi P, Bellone P, Ortolani P, et al. Prognosis of asymptomatic patients with hypertrophic cardiomyopathy and nonsustained ventricular tachycardia. Circulation. 1994;90:2743–7. 7994816 10.1161/01.cir.90.6.2743

[91] Kiaos A, Daskalopoulos GN, Kamperidis V, Ziakas A, Efthimiadis G, Karamitsos TD. Quantitative late gadolinium enhancement cardiac magnetic resonance and sudden death in hypertrophic cardiomyopathy: a meta-analysis. JACC Cardiovasc Imaging. 2024;17:489–97. 37632503 10.1016/j.jcmg.2023.07.005

[92] Choi YJ, Lee HJ, Park JS, Park CS, Rhee TM, Choi JY, et al. Left ventricular global longitudinal strain as a prognosticator in hypertrophic cardiomyopathy with a low-normal left ventricular ejection fraction. Eur Heart J Cardiovasc Imaging. 2023;24:1374–83. 37467475 10.1093/ehjci/jead177

[93] Lee HJ, Kim HK, Lee SC, Kim J, Park JB, Hwang IC, et al. Supplementary role of left ventricular global longitudinal strain for predicting sudden cardiac death in hypertrophic cardiomyopathy. Eur Heart J Cardiovasc Imaging. 2022;23:1108–16. 34542591 10.1093/ehjci/jeab187

[94] Tower-Rader A, Mohananey D, To A, Lever HM, Popovic ZB, Desai MY. Prognostic value of global longitudinal strain in hypertrophic cardiomyopathy: a systematic review of existing literature. JACC Cardiovasc Imaging. 2019;12:1930–42. 30219395 10.1016/j.jcmg.2018.07.016

[95] Al Wazzan AA, Taconne TM, Ribet FR, Le Rolle VL, Hermann Haugaa KH, Edvardsen TE, et al. Identification and validation of ventricular rhythmic risk subgroups in hypertrophic cardiomyopathy by clustering analysis including left ventricular strain analysis. Eur Heart J. 2024;45(Suppl_1):ehae666.036.

[96] Wang J, Zhang J, Pu L, Qi W, Xu Y, Wan K, et al. The prognostic value of left ventricular entropy from T1 mapping in patients with hypertrophic cardiomyopathy. JACC Asia. 2024;4:389–99. 38765656 10.1016/j.jacasi.2024.01.005 PMC11099820

[97] Xu J, Zhuang B, Sirajuddin A, Li S, Huang J, Yin G, et al. MRI T1 mapping in hypertrophic cardiomyopathy: evaluation in patients without late gadolinium enhancement and hemodynamic obstruction. Radiology. 2020;294:275–86. 31769741 10.1148/radiol.2019190651 PMC6996717

[98] Ye Y, Ji Z, Zhou W, Pu C, Li Y, Zhou C, et al. Mean scar entropy by late gadolinium enhancement cardiac magnetic resonance is associated with ventricular arrhythmias events in hypertrophic cardiomyopathy. Front Cardiovasc Med. 2021;8: 758635. 34869672 10.3389/fcvm.2021.758635 PMC8635716

[99] Zhao X, Jin F, Wang J, Zhao X, Wang L, Wei H. Entropy of left ventricular late gadolinium enhancement and its prognostic value in hypertrophic cardiomyopathy a new CMR assessment method. Int J Cardiol. 2023;373:134–41. 36395920 10.1016/j.ijcard.2022.11.017

[100] Wu CW, Wu R, Shi RY, An DA, Chen BH, Jiang M, et al. Histogram analysis of native T mapping and its relationship to left ventricular late gadolinium enhancement, hypertrophy, and segmental myocardial mechanics in patients with hypertrophic cardiomyopathy. J Magn Reson Imaging. 2019;49:668–77. 30142234 10.1002/jmri.26272

[101] Coppini R, Ho CY, Ashley E, Day S, Ferrantini C, Girolami F, et al. Clinical phenotype and outcome of hypertrophic cardiomyopathy associated with thin-filament gene mutations. J Am Coll Cardiol. 2014;64:2589–600. 25524337 10.1016/j.jacc.2014.09.059 PMC4270453

[102] Varnava AM, Elliott PM, Baboonian C, Davison F, Davies MJ, McKenna WJ. Hypertrophic cardiomyopathy: histopathological features of sudden death in cardiac troponin T disease. Circulation. 2001;104:1380–4. 11560853 10.1161/hc3701.095952

[103] Shafaattalab S, Li AY, Gunawan MG, Kim B, Jayousi F, Maaref Y, et al. Mechanisms of arrhythmogenicity of hypertrophic cardiomyopathy-associated troponin T () variant I79N. Front Cell Dev Biol. 2021;9: 787581. 34977031 10.3389/fcell.2021.787581 PMC8718794

[104] Sedaghat-Hamedani F, Kayvanpour E, Tugrul OF, Lai A, Amr A, Haas J, et al. Clinical outcomes associated with sarcomere mutations in hypertrophic cardiomyopathy: a meta-analysis on 7675 individuals. Clin Res Cardiol. 2018;107:30–41. 28840316 10.1007/s00392-017-1155-5

[105] Magrì D, Limongelli G, Re F, Agostoni P, Zachara E, Correale M, et al. Cardiopulmonary exercise test and sudden cardiac death risk in hypertrophic cardiomyopathy. Heart. 2016;102:602–9. 26849900 10.1136/heartjnl-2015-308453

[106] Sugrue A, Killu AM, DeSimone CV, Chahal AA, Vogt JC, Kremen V, et al. Utility of T-wave amplitude as a non-invasive risk marker of sudden cardiac death in hypertrophic cardiomyopathy. Open Heart. 2017;4: e000561. 28409011 10.1136/openhrt-2016-000561 PMC5384475

[107] Biagini E, Pazzi C, Olivotto I, Musumeci B, Limongelli G, Boriani G, et al. Usefulness of electrocardiographic patterns at presentation to predict long-term risk of cardiac death in patients with hypertrophic cardiomyopathy. Am J Cardiol. 2016;118:432–9. 27289293 10.1016/j.amjcard.2016.05.023

[108] Debonnaire P, Katsanos S, Joyce E, VAN DEN Brink OV, Atsma DE, Schalij MJ, et al. QRS fragmentation and QTc duration relate to malignant ventricular tachyarrhythmias and sudden cardiac death in patients with hypertrophic cardiomyopathy. J Cardiovasc Electrophysiol. 2015;26:547–55. 10.1111/jce.12629 25648421

[109] Park YM. Updated risk assessments for sudden cardiac death in hypertrophic cardiomyopathy patients with implantable cardioverter-defibrillator. Korean J Intern Med. 2023;38:7–15. 36353786 10.3904/kjim.2022.144 PMC9816680

[110] Akboğa MK, Gülcihan Balcı K, Yılmaz S, Aydın S, Yayla Ç, Ertem AG, et al. Tp-e interval and Tp-e/QTc ratio as novel surrogate markers for prediction of ventricular arrhythmic events in hypertrophic cardiomyopathy. Anatol J Cardiol. 2017;18:48–53. 28315570 10.14744/AnatolJCardiol.2017.7581 PMC5512198

[111] Wu G, Liu J, Wang S, Yu S, Zhang C, Wang D, et al. N-terminal pro-brain natriuretic peptide and sudden cardiac death in hypertrophic cardiomyopathy. Heart. 2021;107:1576–83. 33361398 10.1136/heartjnl-2020-317701

[112] Kim K, Lee SD, Lee HJ, Kim H, Kim HR, Cho YH, et al. Role and clinical importance of progressive changes in echocardiographic parameters in predicting outcomes in patients with hypertrophic cardiomyopathy. J Cardiovasc Imaging. 2023;31:85–95. 37096673 10.4250/jcvi.2022.0053 PMC10133807

[113] Teare D. Asymmetrical hypertrophy of the heart in young adults. Br Heart J 1958;20:1-8 10.1136/hrt.20.1.1 PMC492780 13499764

